# Supplementary material for: Synthesis of Galectin Inhibitors by Regioselective 3′-O-Sulfation of Vanillin Lactosides Obtained under Phase Transfer Catalysis
Source: Molecules. 2020 Dec 29;26(1):115. doi: 10.3390/molecules26010115 (PMC7795656; doi:10.3390/molecules26010115)
Supplement: Supplementary file 1 [file molecules-26-00115-s001.pdf]

SUPPLEMENTARY MATERIALS

Synthesis of Galectins Inhibitors by Regioselective 3'-O-Sulfation of Vanillin Lactosides Obtained Under Phase Transfer Catalysis

Karima Belkhadem[1], Yihong Cao[1] and René Roy[1,2,*]

[1] Department of Chemistry, University of Québec a Montréal, P.O. Box 8888, Succ. Centre-Ville, Montréal, Québec H3C 3P8, Canada, E-mail : roy.rene@uqam.ca

[2] INRS-Institut Armand-Frappier, Université du Québec, 531 boul. des Prairies, Laval, Québec, H7V 1B7, Canada

* Correspondence: roy.rene@uqam.ca; Tel.: +1 514-987-3000, ext. 2546

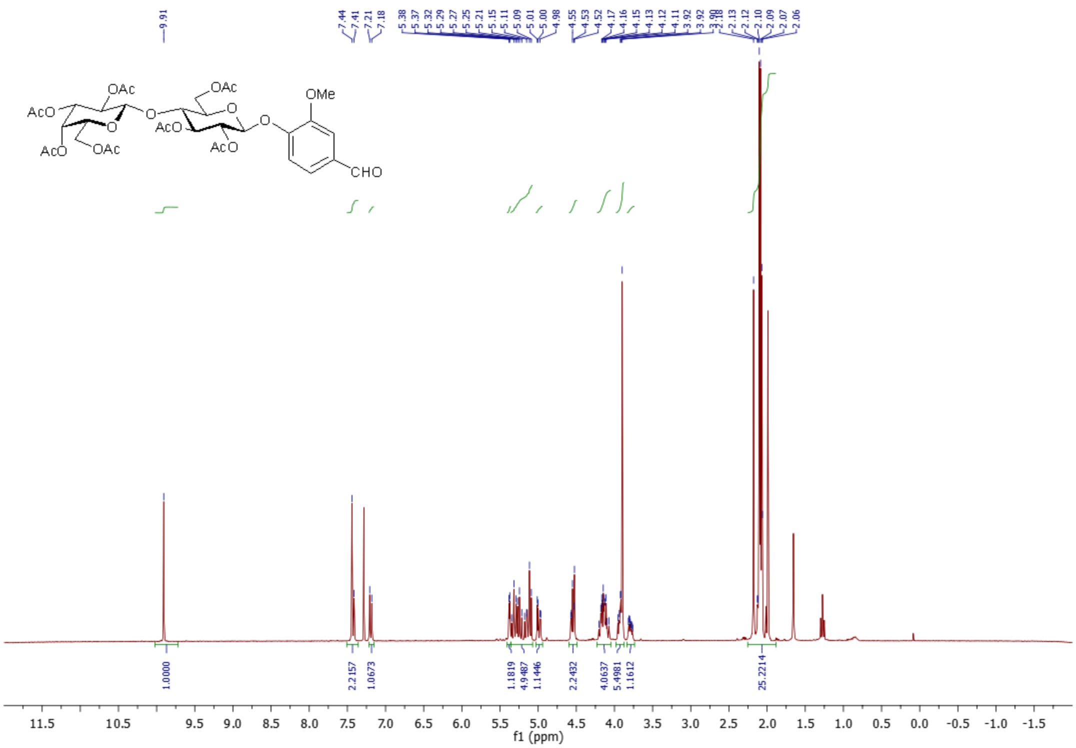

^{1}H NMR spectrum (300 MHz, CDCl3) of compound **5**

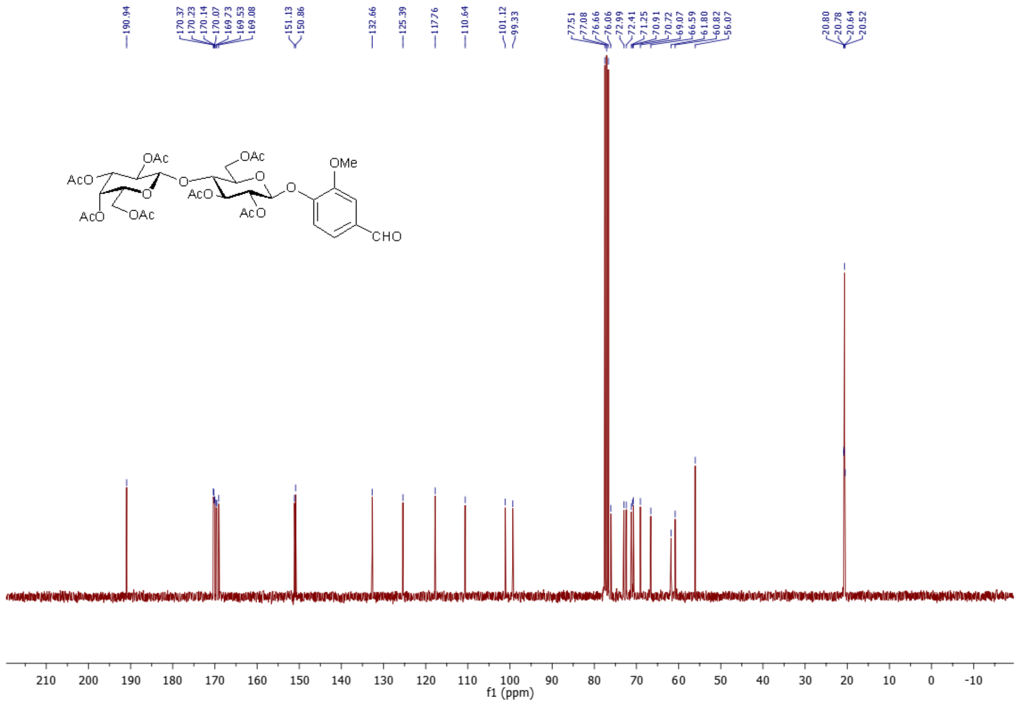

^{13}C NMR spectrum (75 MHz, CDCL3) of compound **5**

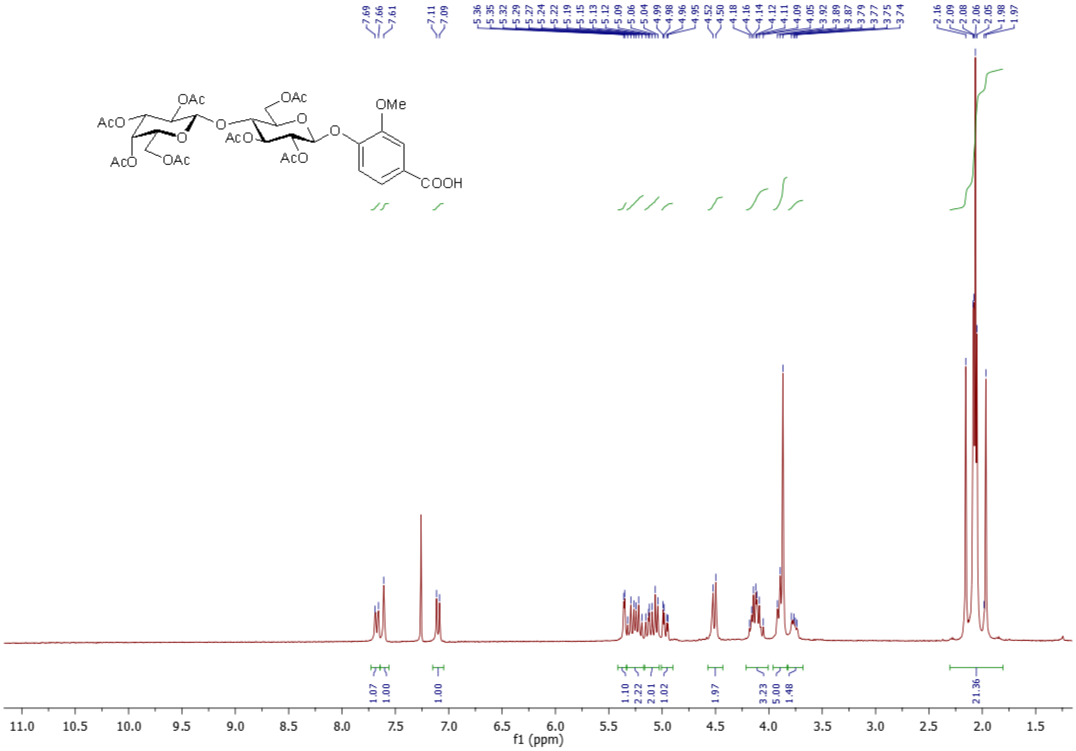

^{1}H NMR spectrum (300 MHz, CDCl3) of compound **6**

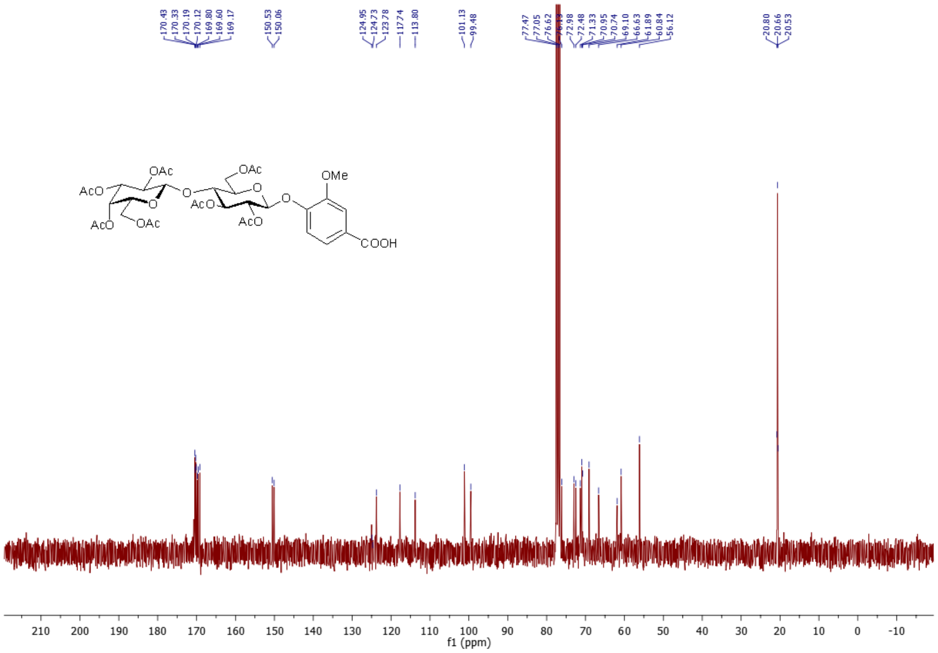

^{13}C NMR spectrum (75 MHz, CDCL3) of compound **6.**

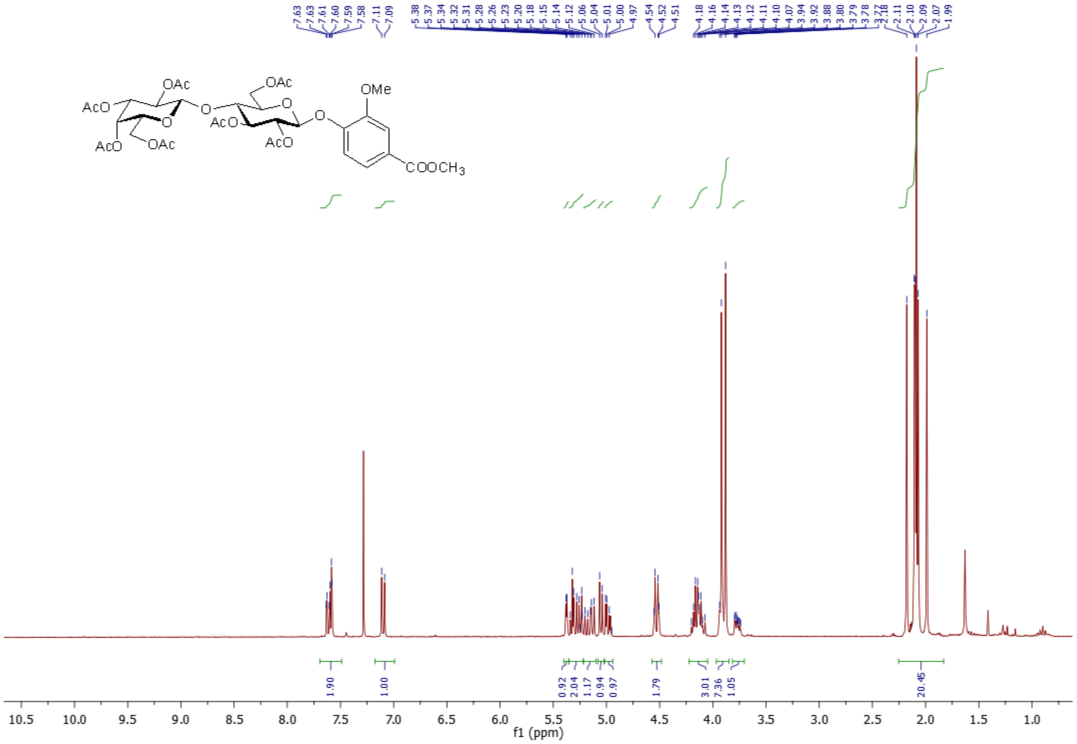

^{1}H NMR spectrum (300 MHz, CDCl3) of compound **7**

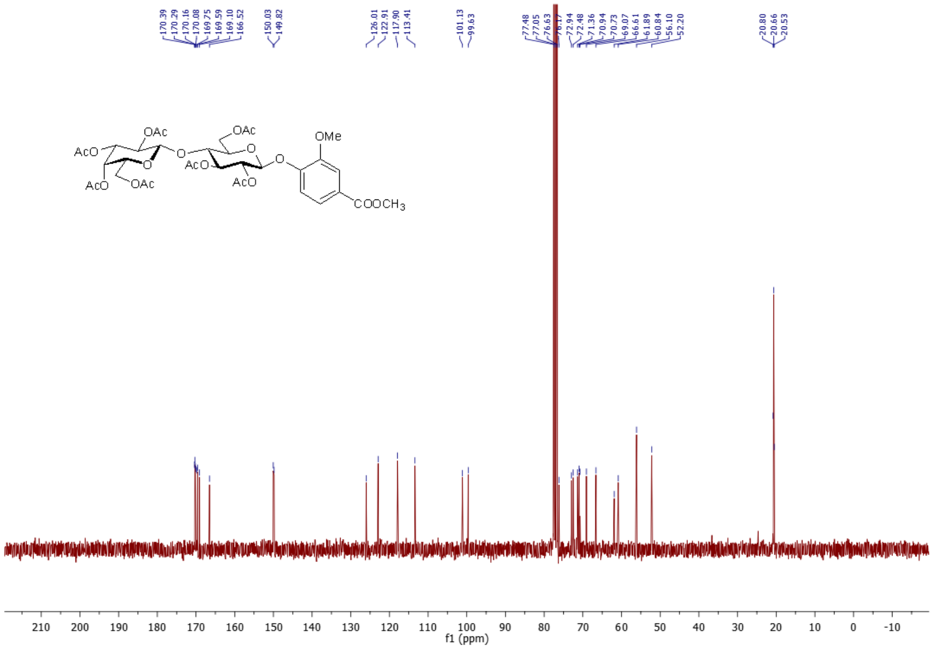

^{13}C NMR spectrum (75 MHz, CDCL3) of compound **7**.

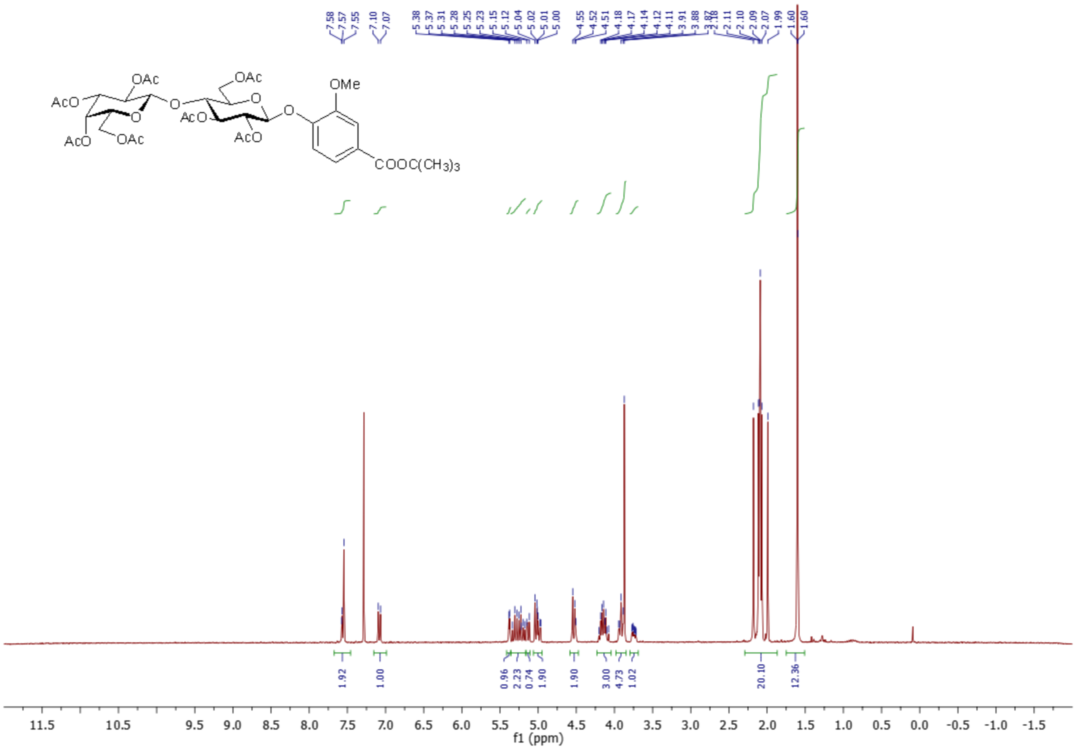

^{1}H NMR spectrum (300 MHz, CDCl3) of compound **8**

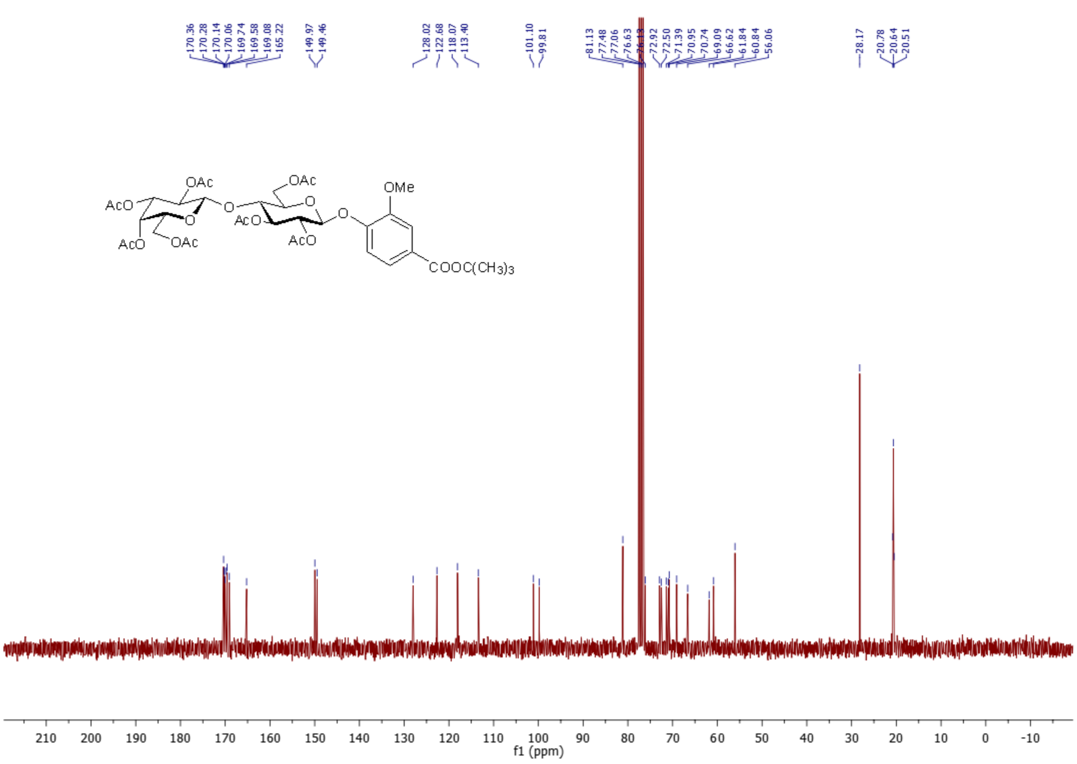

^{13}C NMR spectrum (75 MHz, CDCL3) of compound **8**

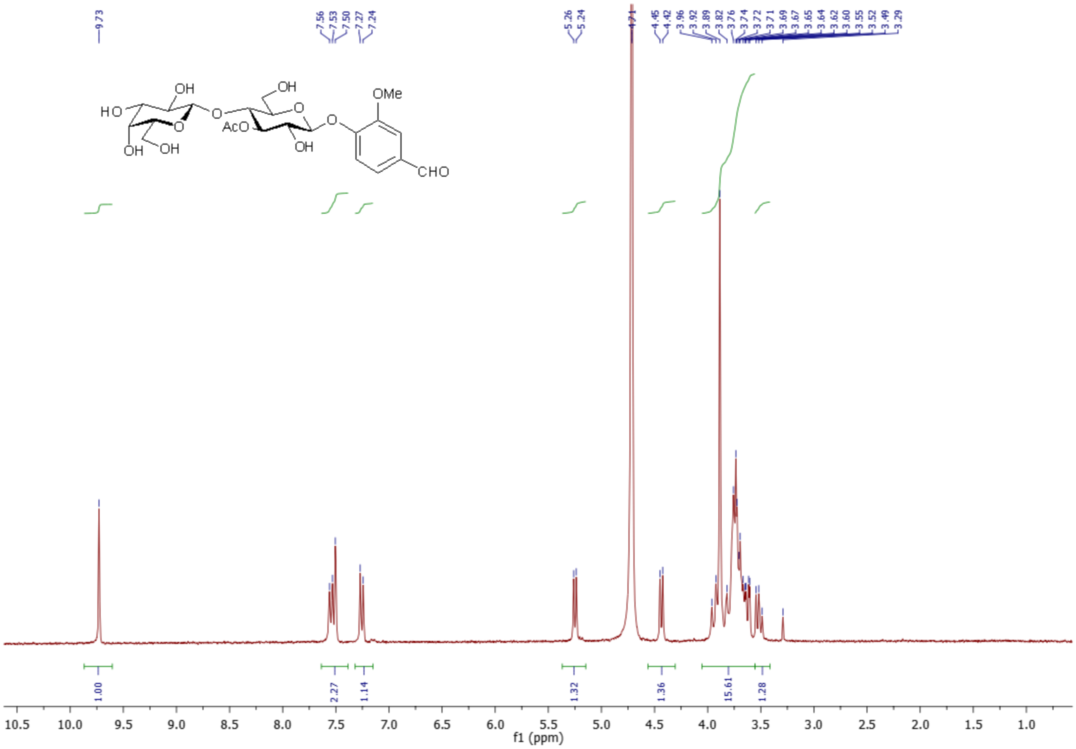

1H NMR spectrum (300 MHz, D2O) of compound **9**.

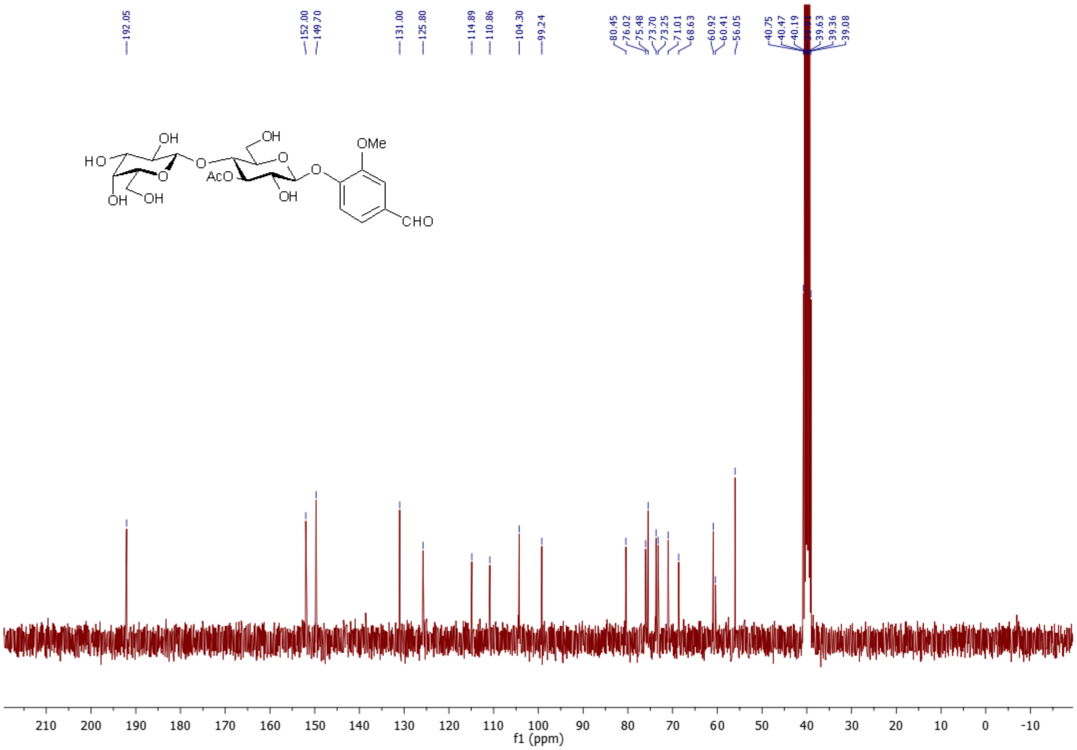

^{13}C NMR spectrum (75MHz, DMSO-d6) of compound **9**.

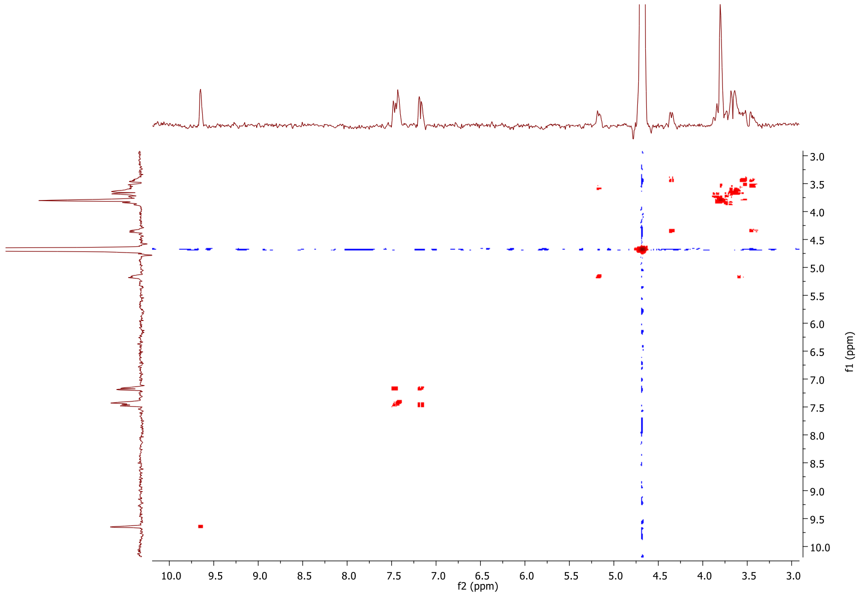

COSY NMR spectrum (300 MHz, D2O) of compound **9**.

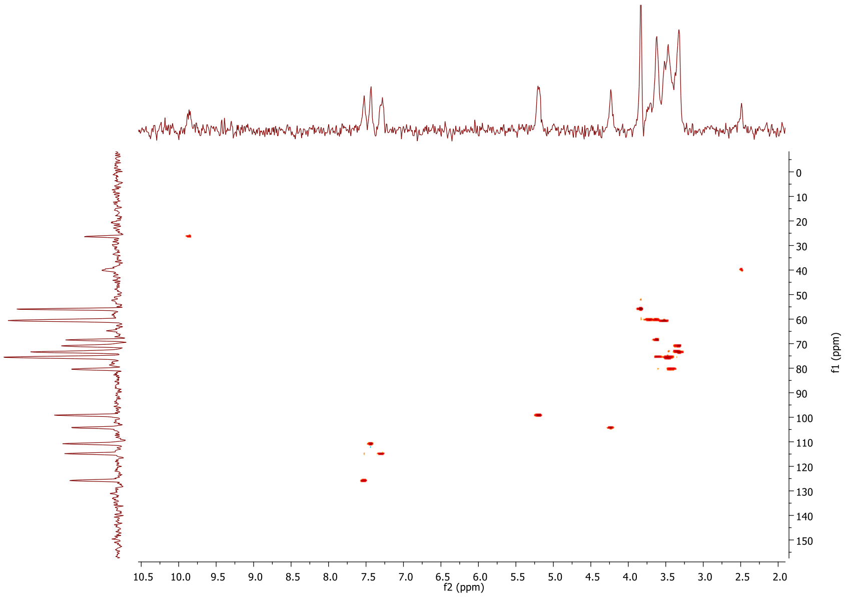

HSQC NMR spectrum (75 MHz, DMSO-d6) of compound **9**.

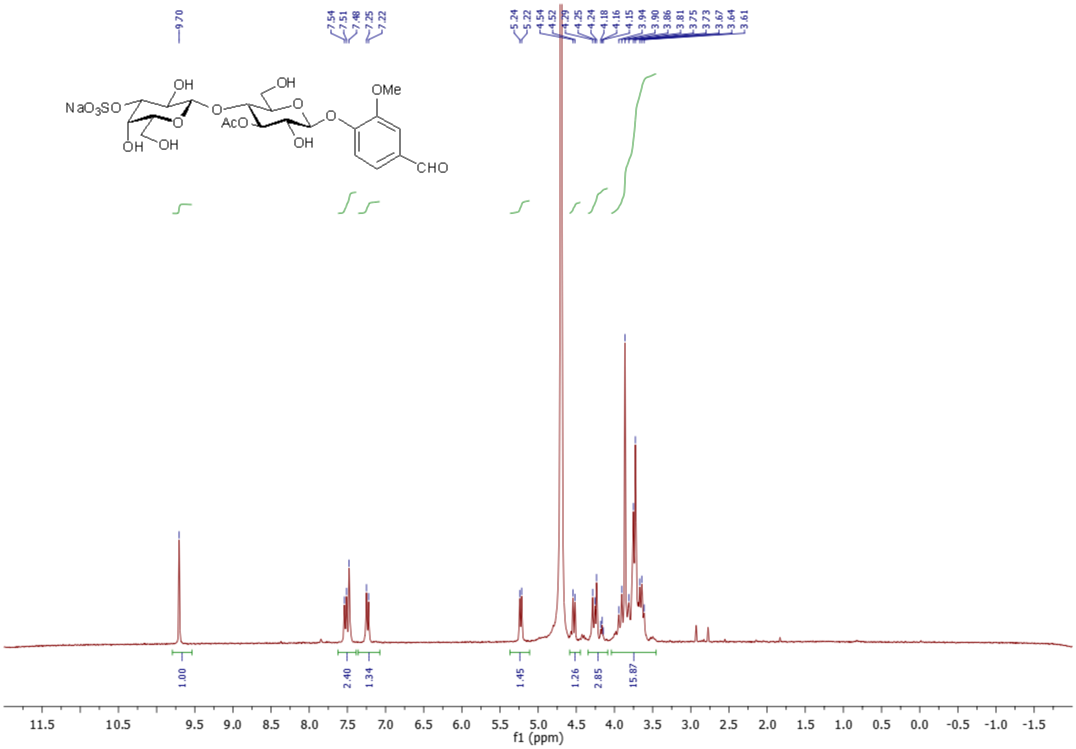

1H NMR spectrum (300 MHz, D_2O) of compound **10**.

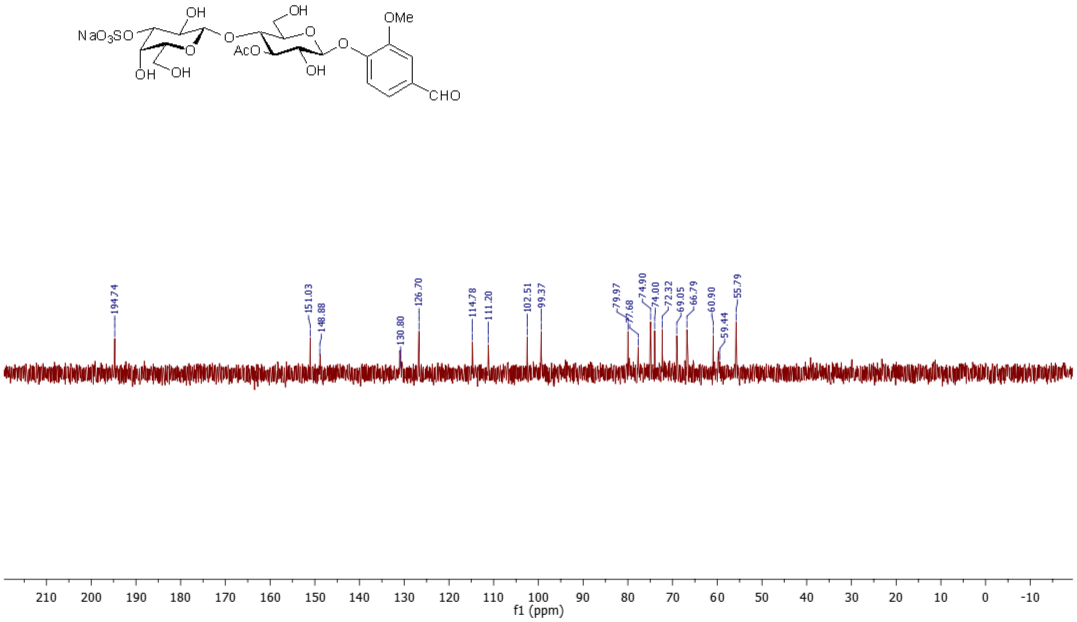

^{13}C NMR spectrum (75MHz, D_2O) of compound **10**.

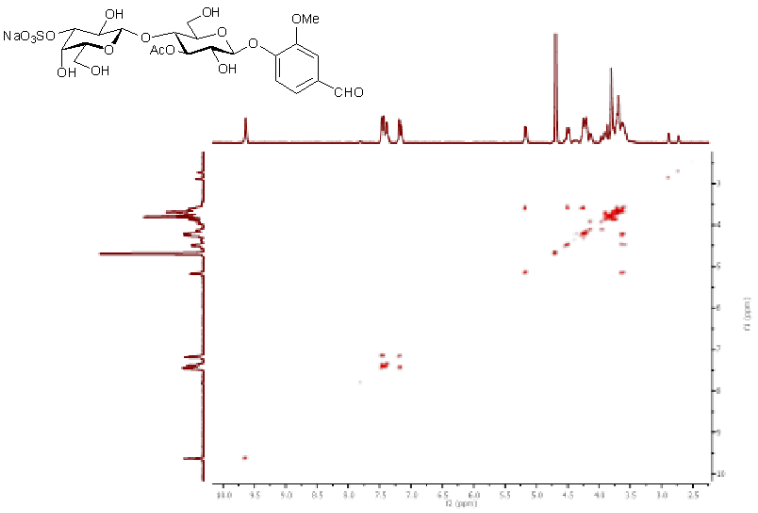

COSY NMR spectrum (300 MHz, D_2O) of compound **10**.

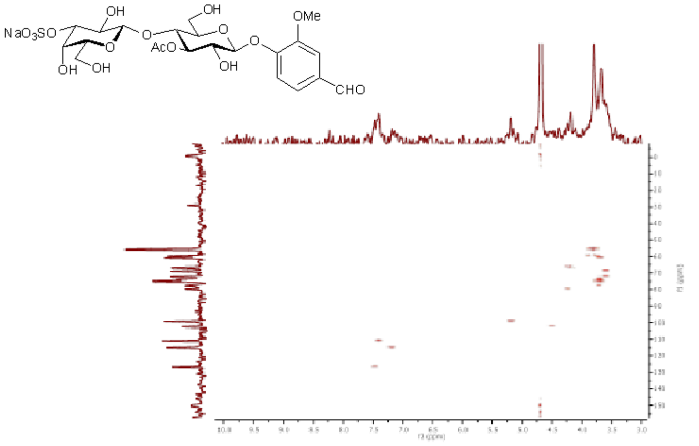

HSQC NMR spectrum (75 MHz, D_2O) of compound **10**.

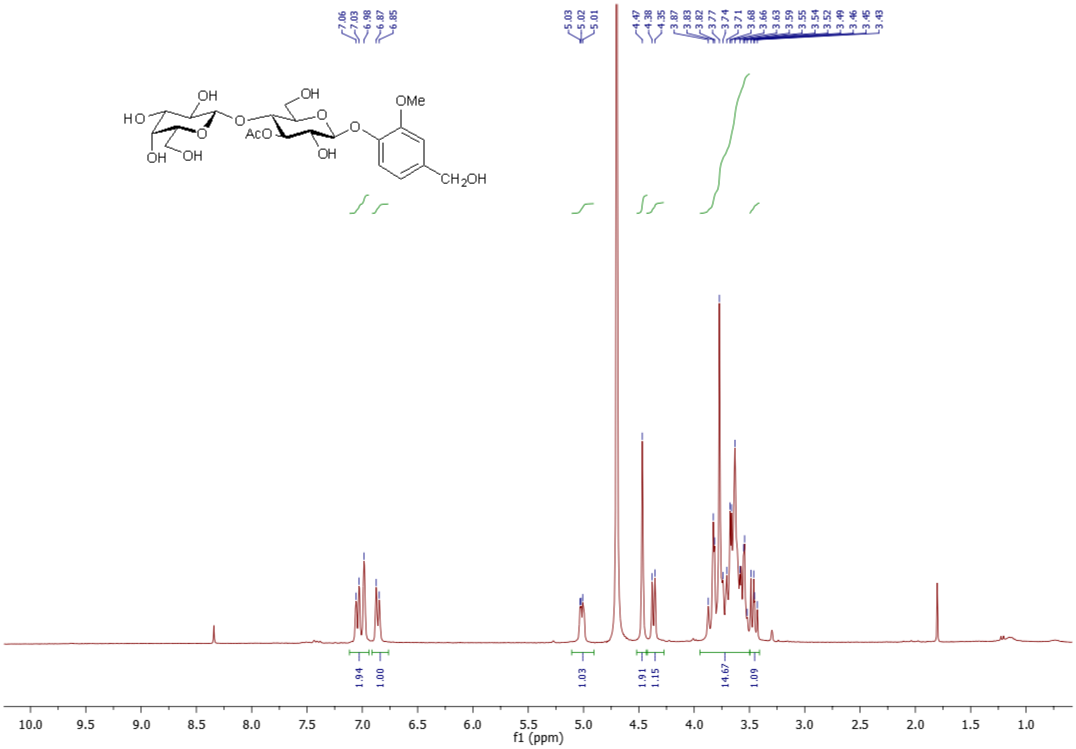

^{1}H NMR spectrum (300 MHz, D_2O) of compound **11**.

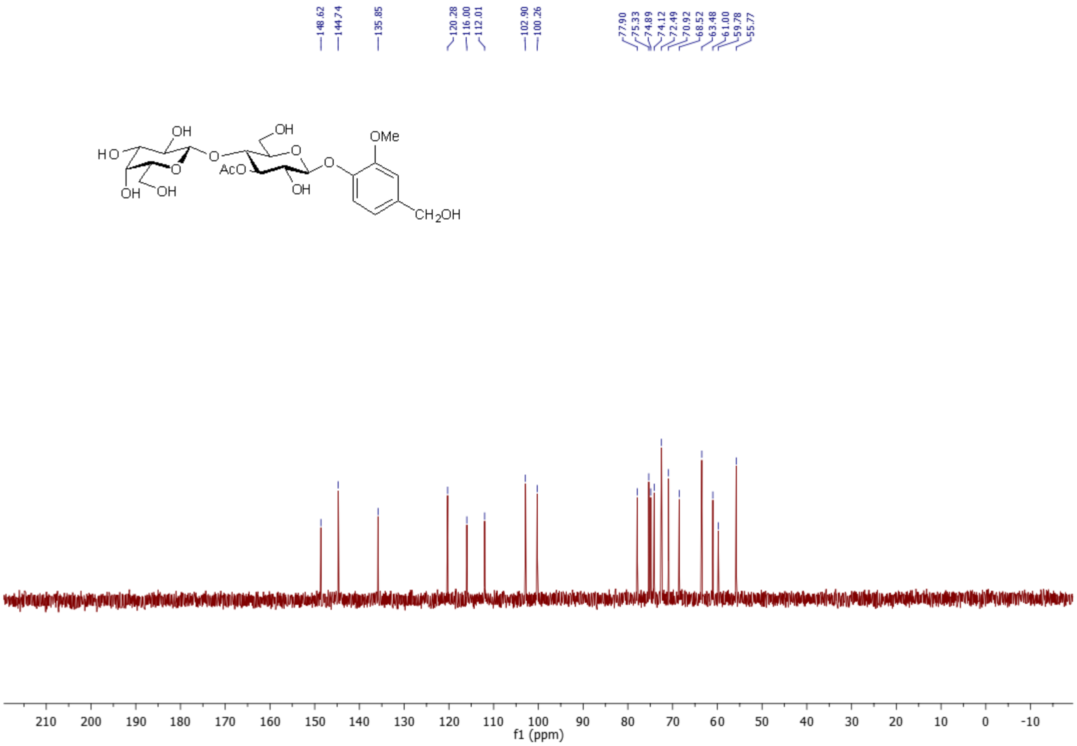

^{13}C NMR spectrum (75 MHz, D_2O) of compound **11**.

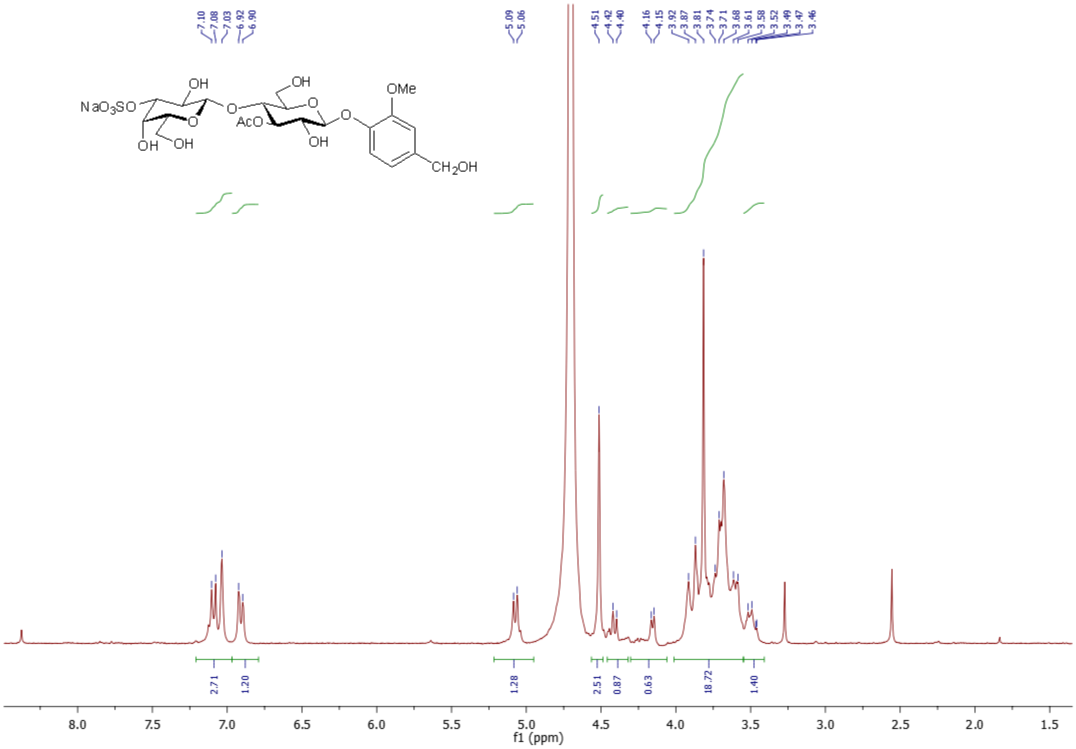

^{1}H NMR spectrum (300 MHz, D_2O) of compound **12**.

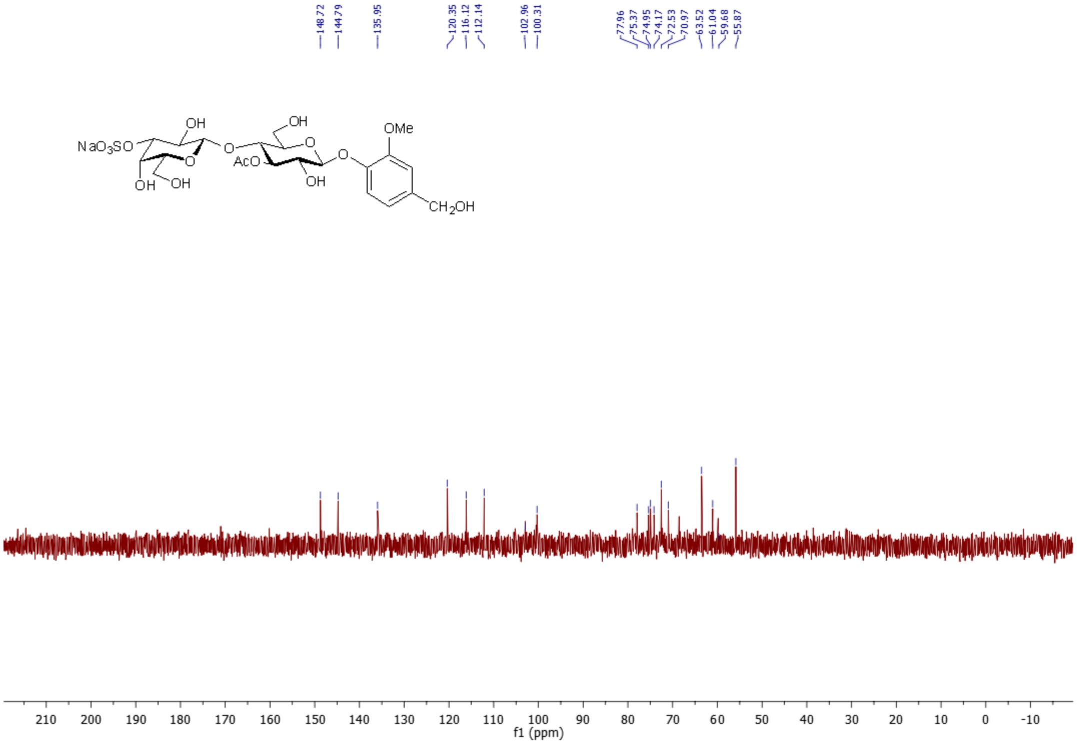

^{13}C NMR spectrum (75 MHz, D_2O) of compound **12**.

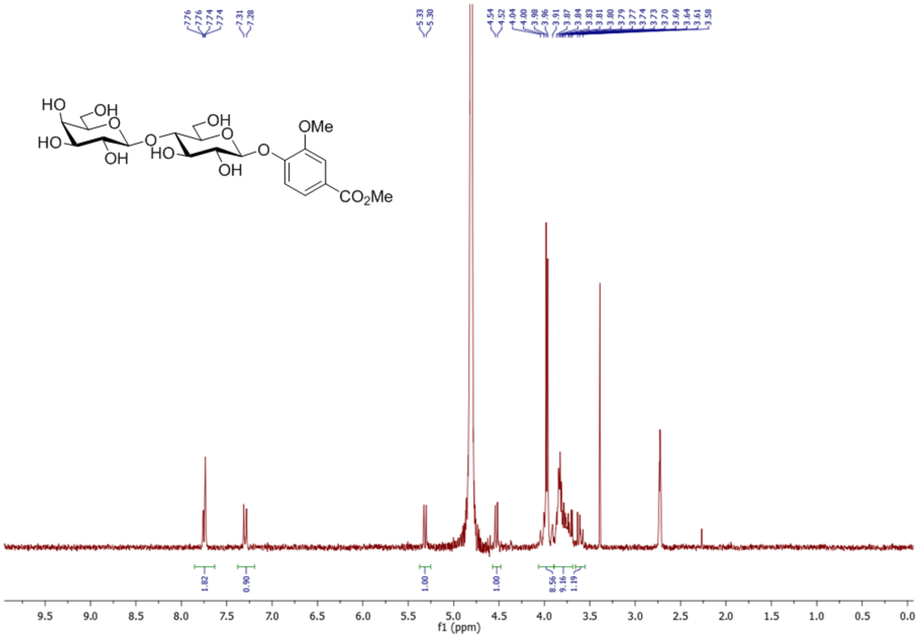

^{1}H NMR spectrum (300 MHz, D_2O+DMSO-d_6) of compound **13**

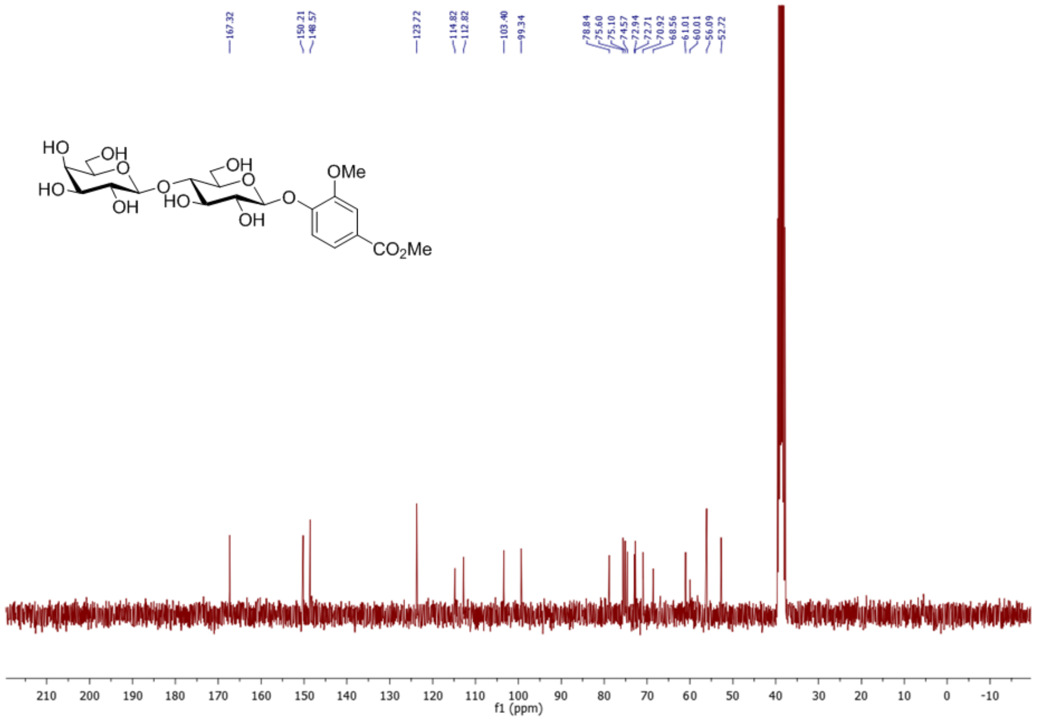

^{13}C NMR spectrum (75 MHz, D_2O+DMSO-d_6) of compound **13**

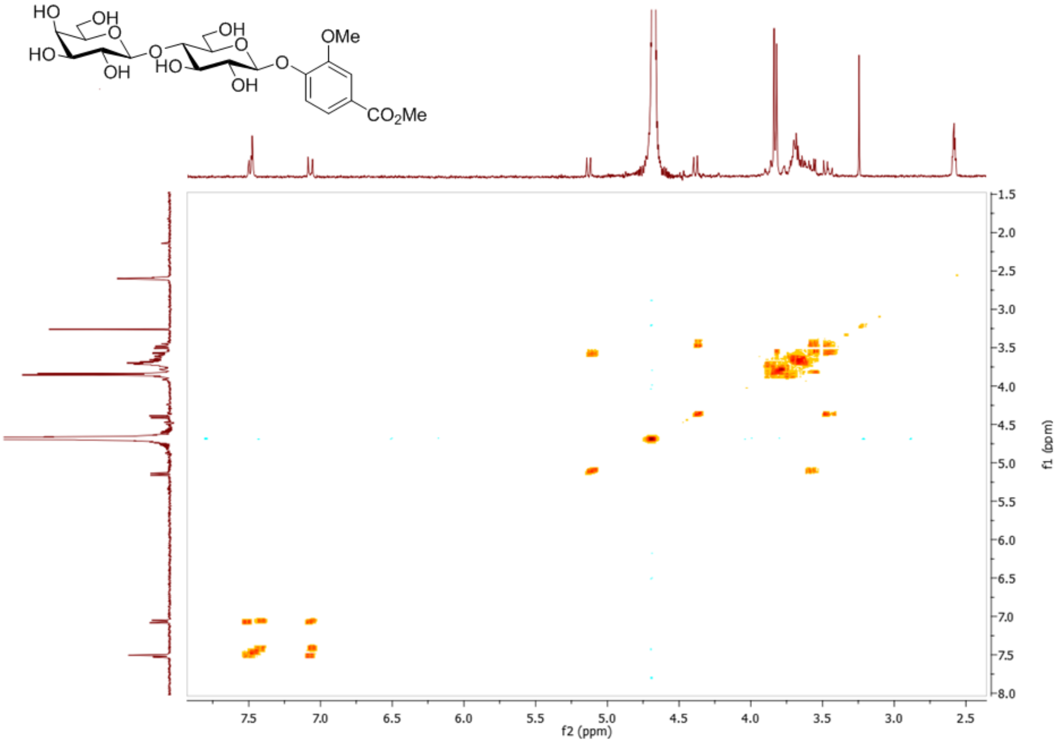

COSY NMR spectrum (D_2O+DMSO-d_6) of compound **13**

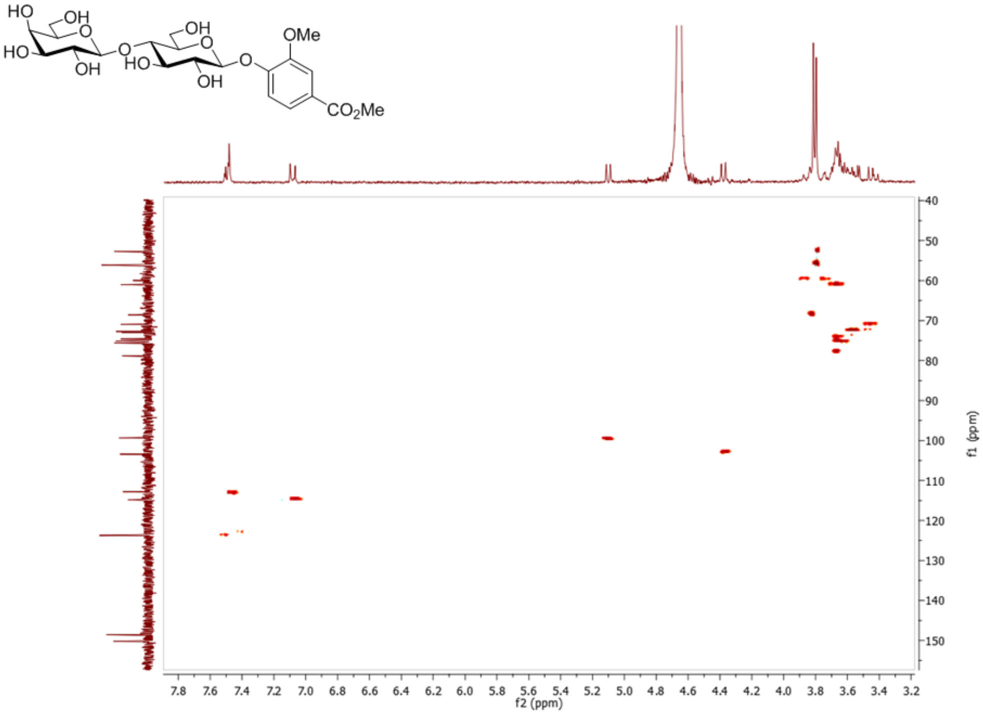

HSQC

HSQC NMR spectrum (D_2O+DMSO-d_6) of compound **13**

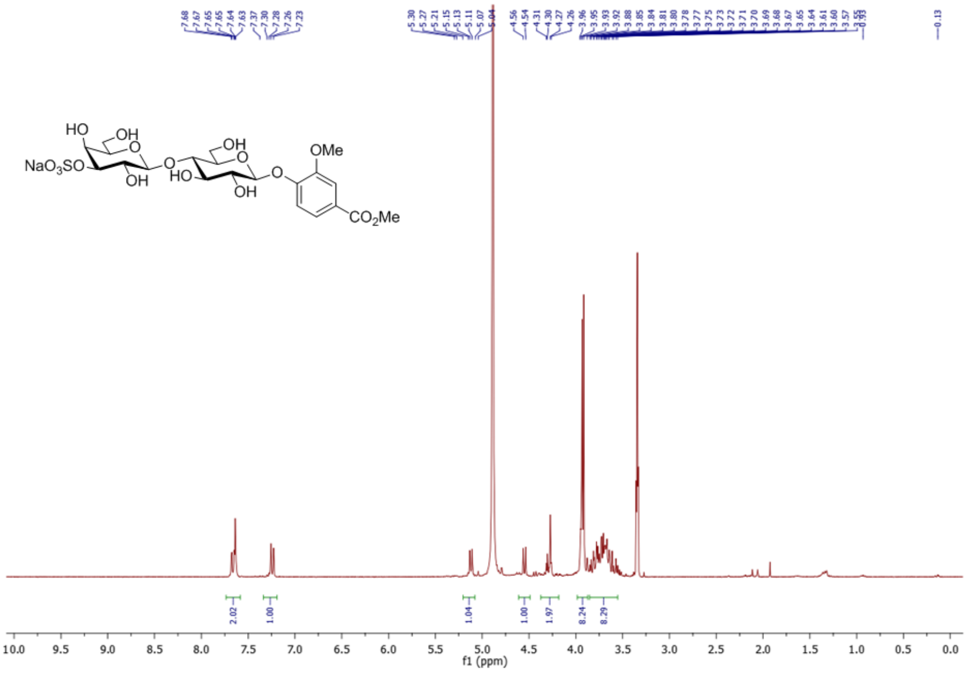

^{1}H NMR spectrum (300 MHz, MeOD) of compound **14**

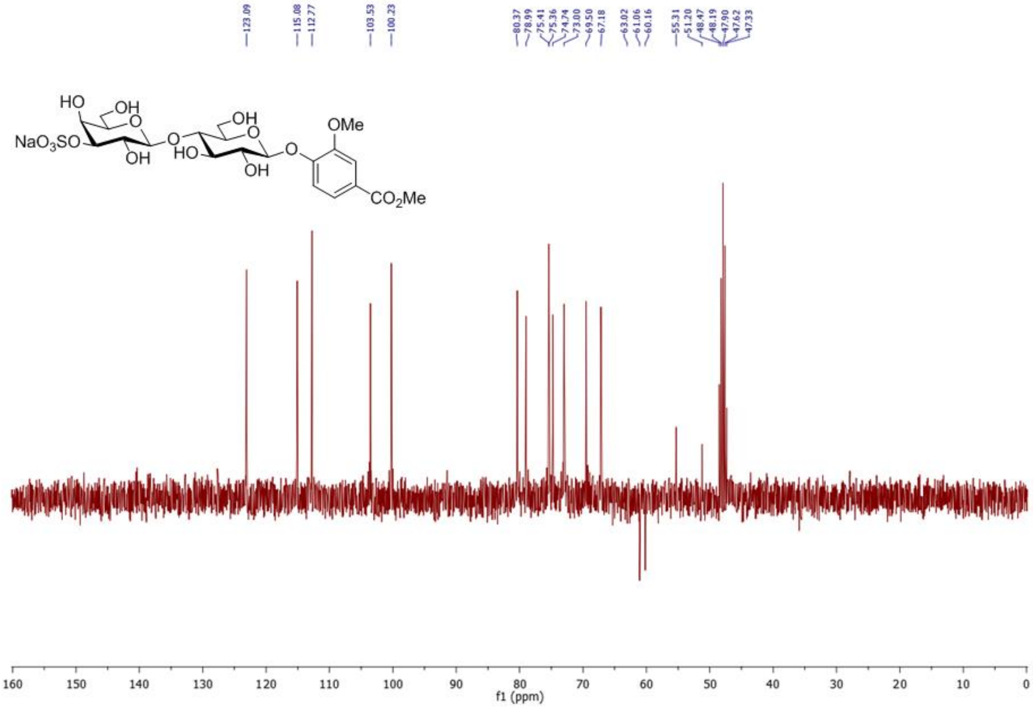

^{13}C Dept135 NMR spectrum (75 MHz, MeOD) of compound **14**

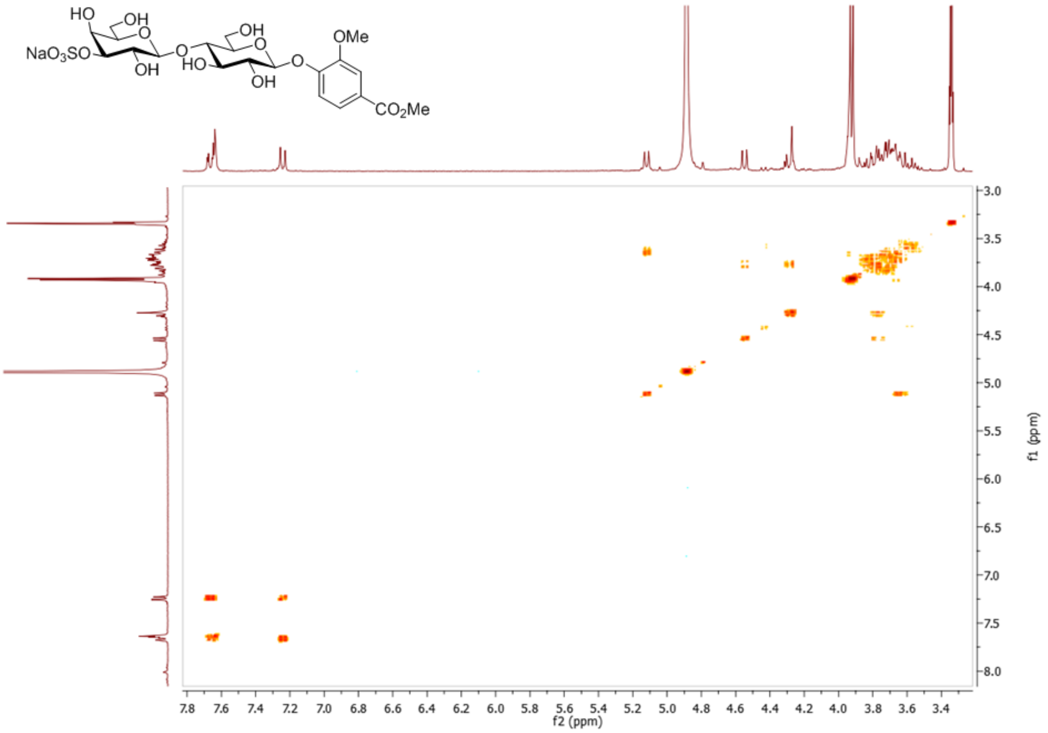

COSY NMR spectrum (MeOD) of compound **14**

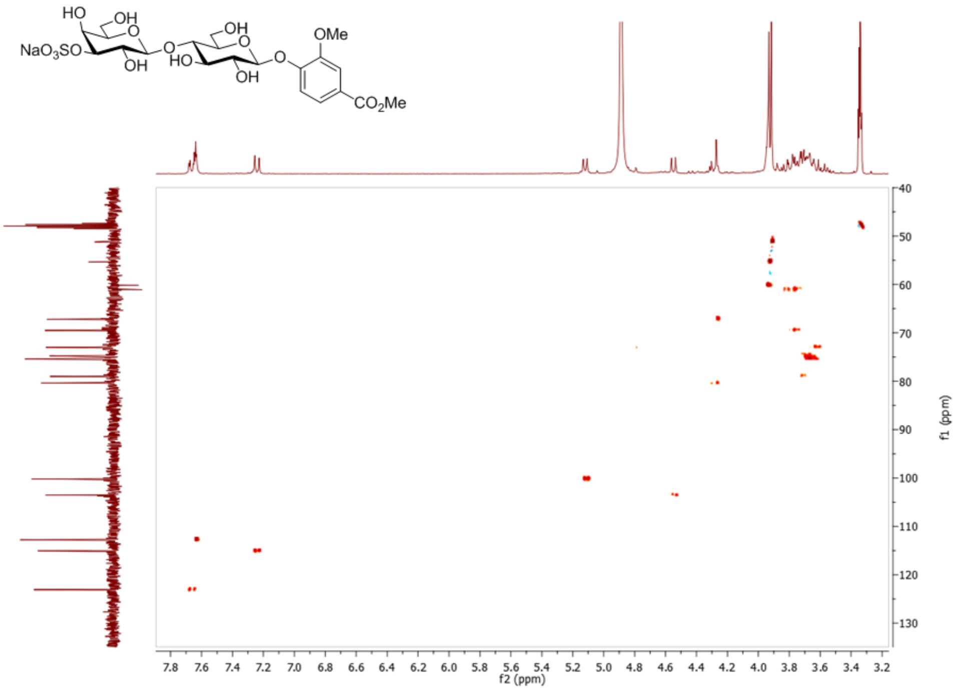

HSQC NMR spectrum (MeOD) of compound **14**

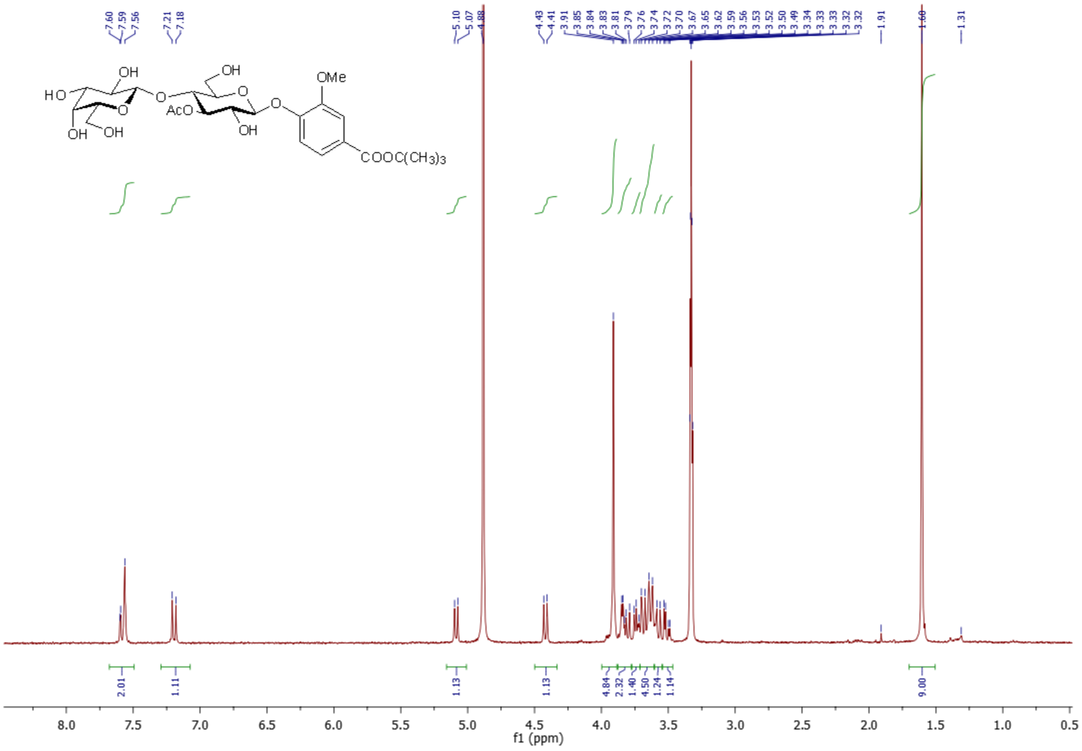

^{1}H NMR spectrum (300 MHz, D_2O) of compound **15**.

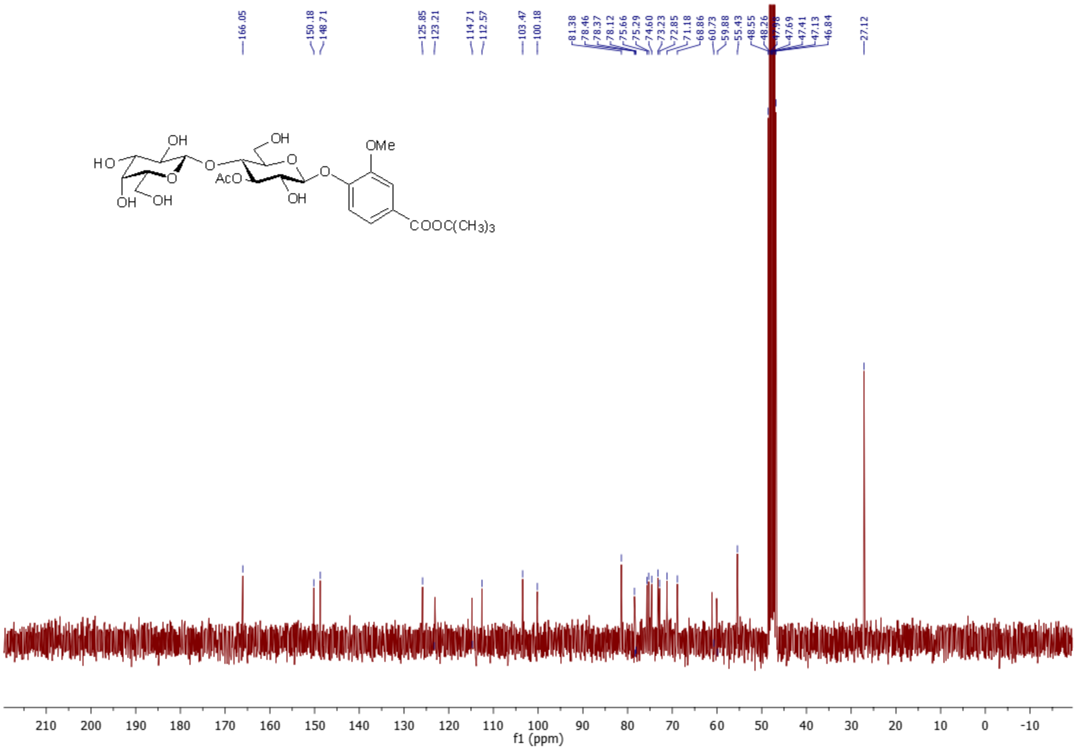

^{13}C NMR spectrum (75 MHz, CD_3OD-d_4) of compound **15**

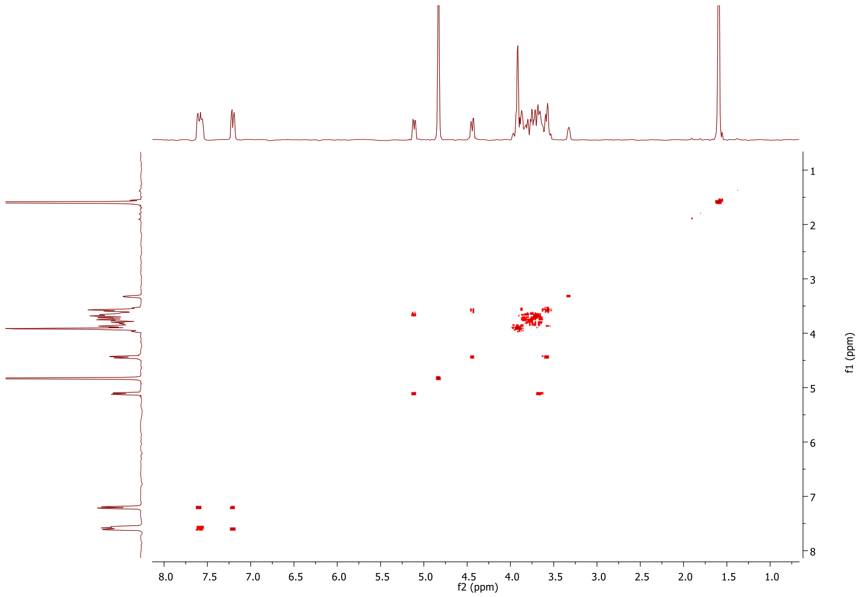

COSY NMR spectrum (300 MHz, D_2O) of compound **15**.

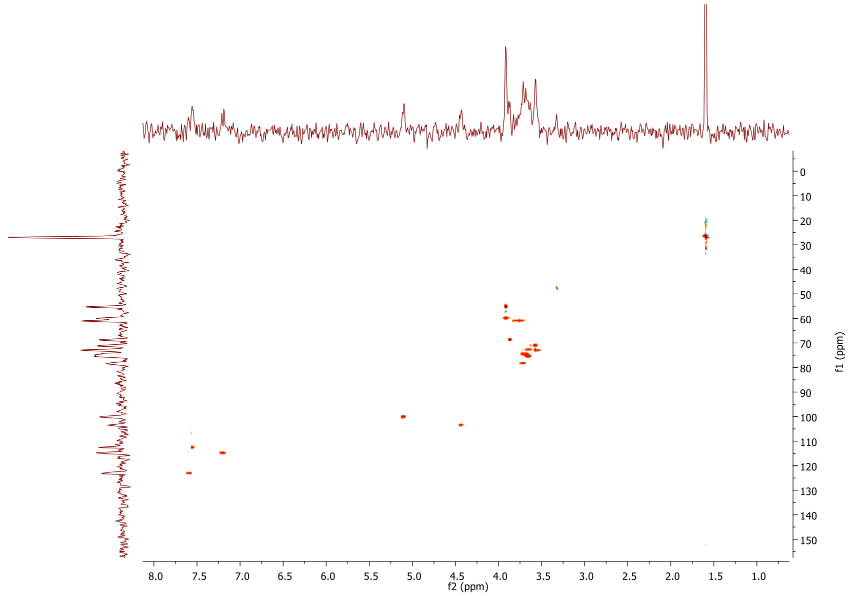

HSQC NMR spectrum (75 MHz, D_2O) of compound **15**.

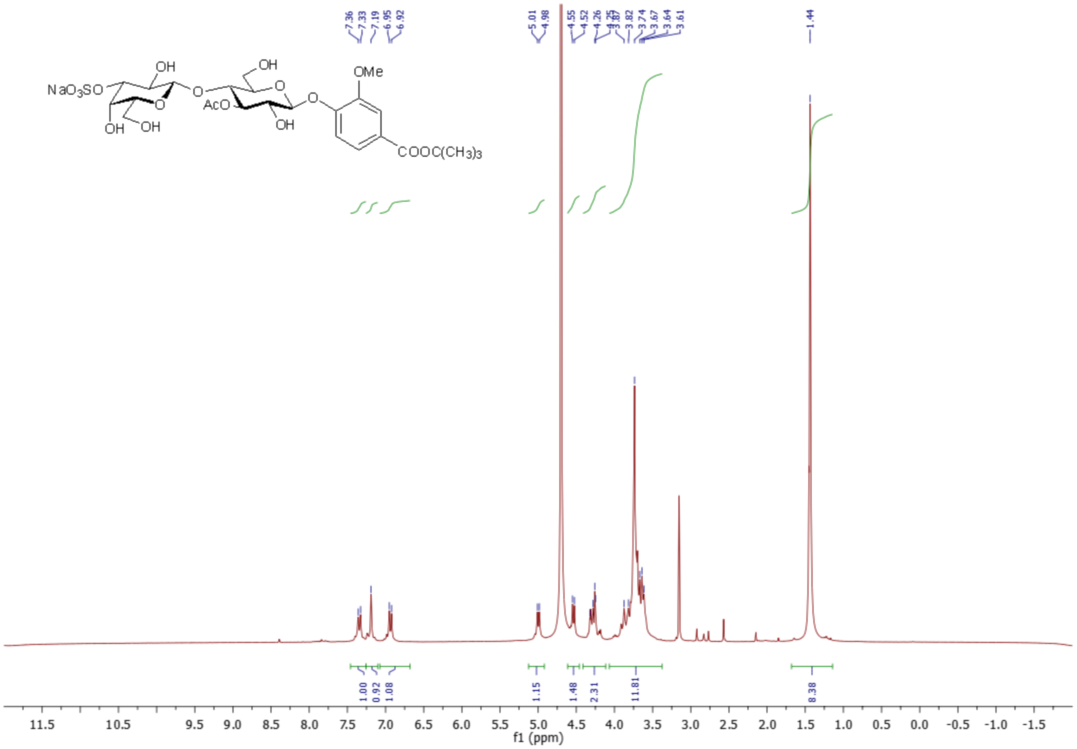

^{1}H NMR spectrum (300 MHz, CD_3OD-d_4) of compound **16**.

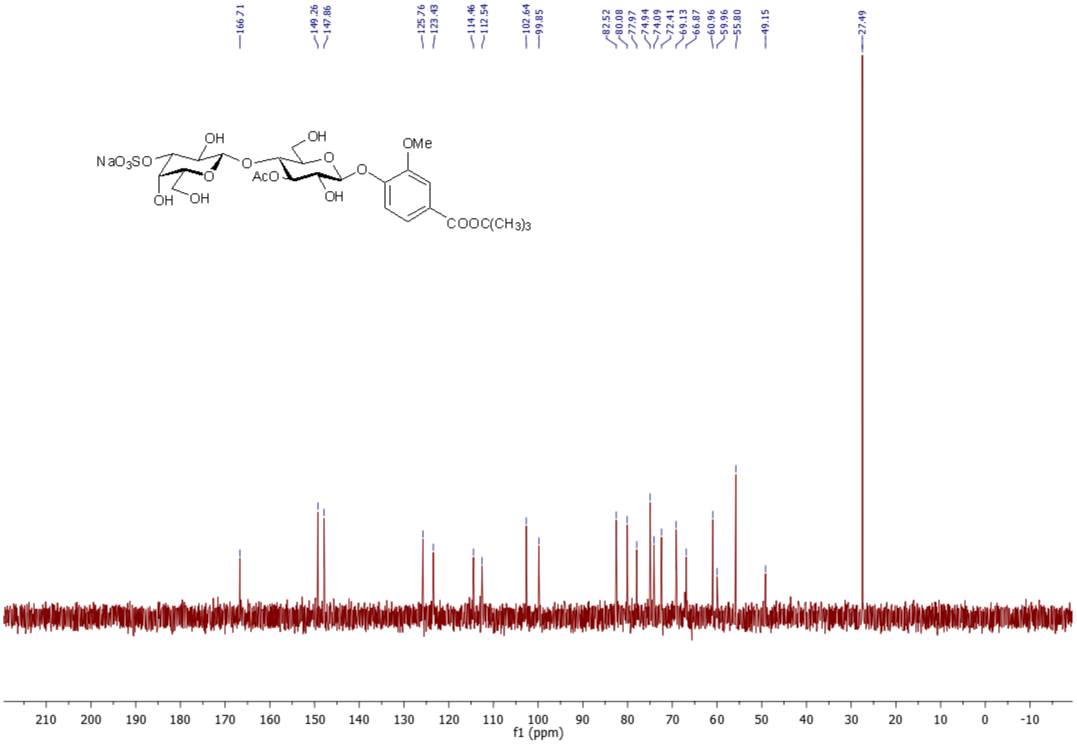

^{13}C NMR spectrum (75 MHz, CD_3OD-d_4) of compound **16**

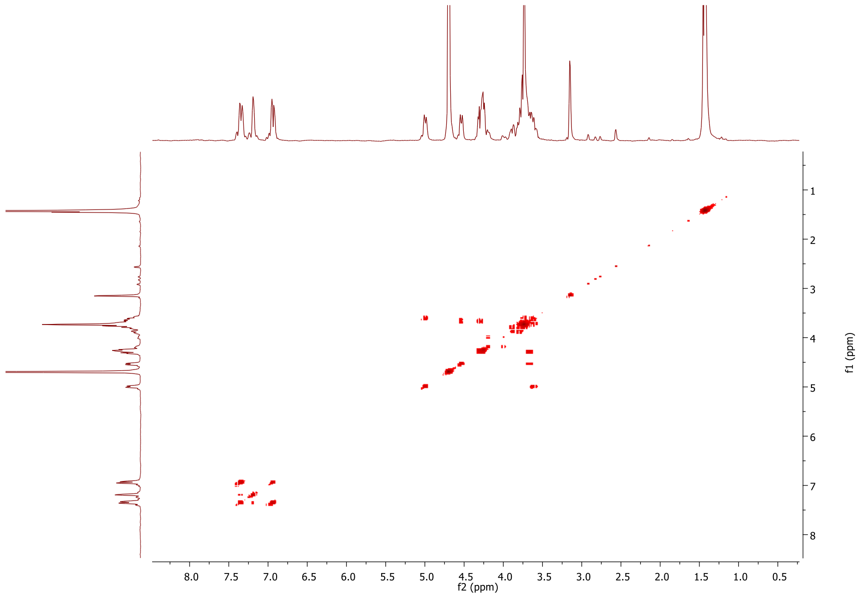

COSY NMR spectrum (300 MHz, CD_3OD-d_4) of compound **16**.

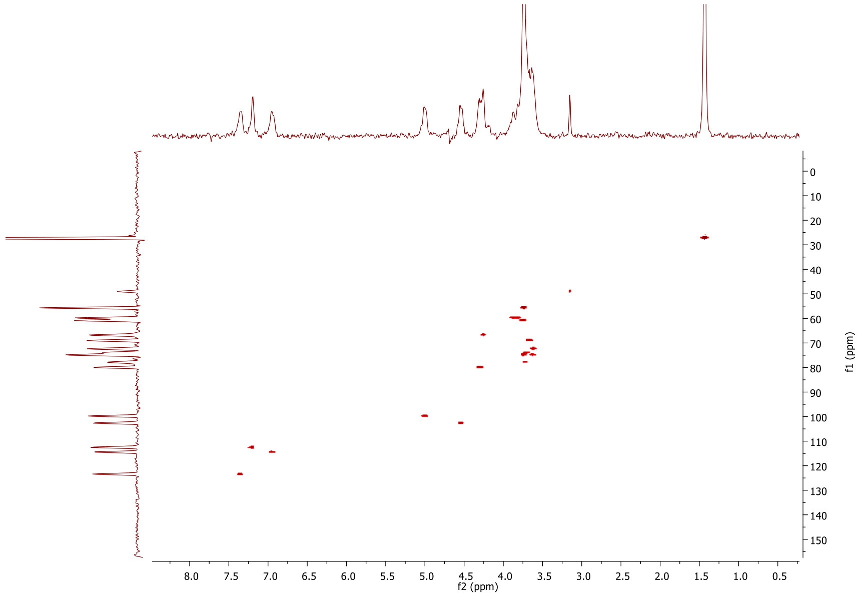

HSQC NMR spectrum (75 MHz, CD_3OD-d_4) of compound **16**.

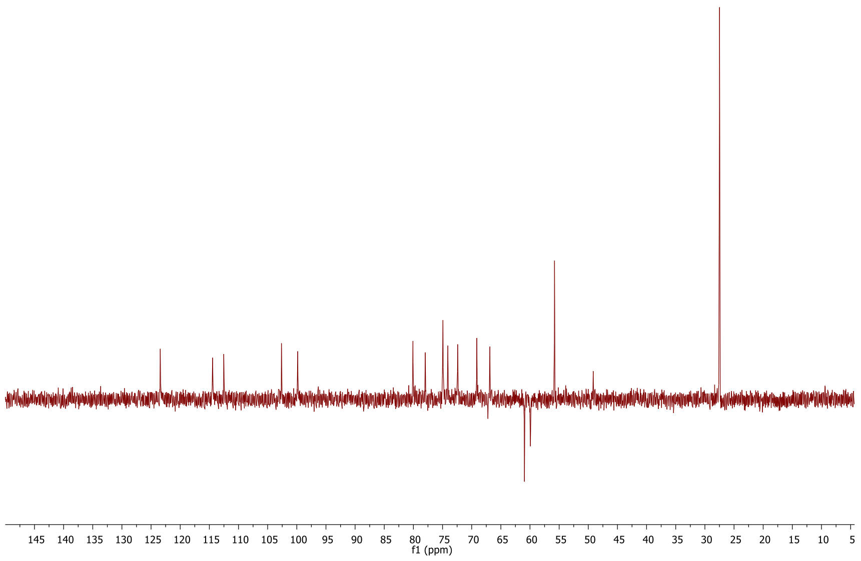

^{13}C Dept135 NMR spectrum (75 MHz, CD_3OD-d_4) of compound **16**.

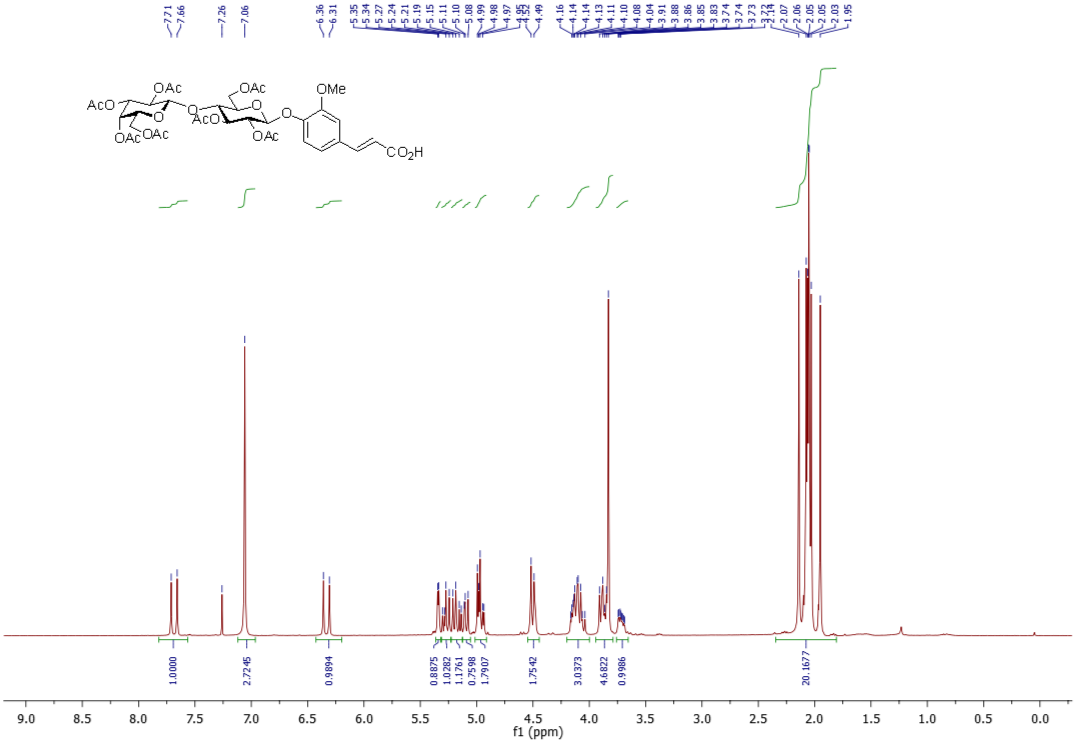

^{1}H NMR spectrum (300 MHz, $CDCl_3$) of compound **17**.

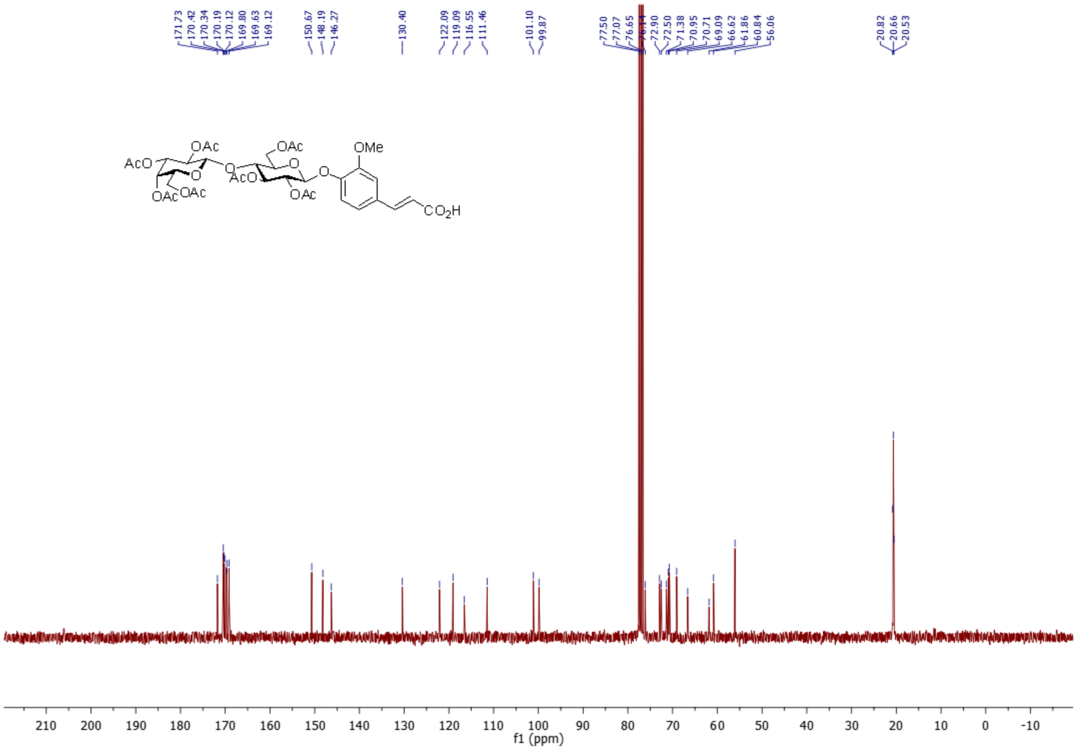

^{13}C NMR spectrum (75MHz, $CDCl_3$) of compound **17**.

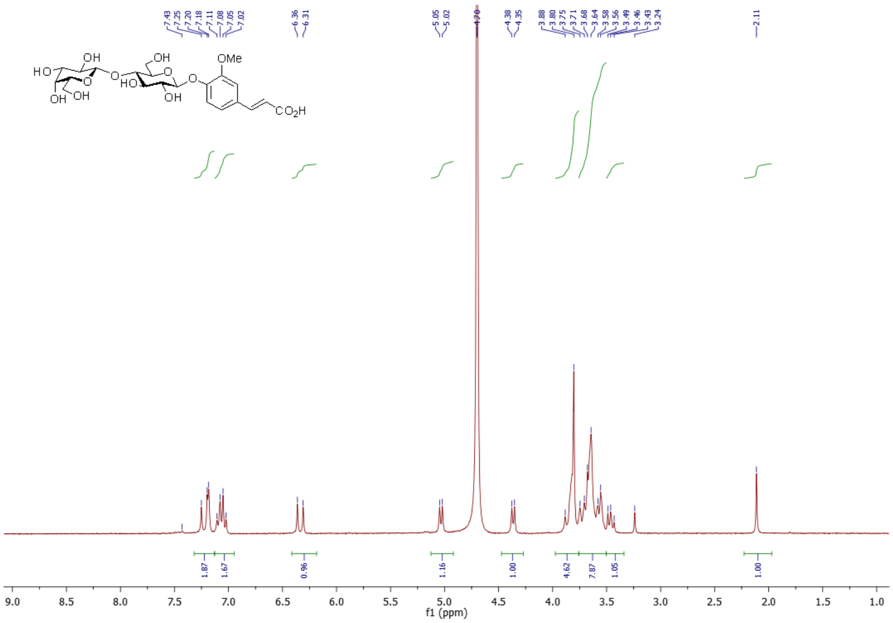

^{1}H NMR spectrum (300 MHz, D_2O) of compound **18**

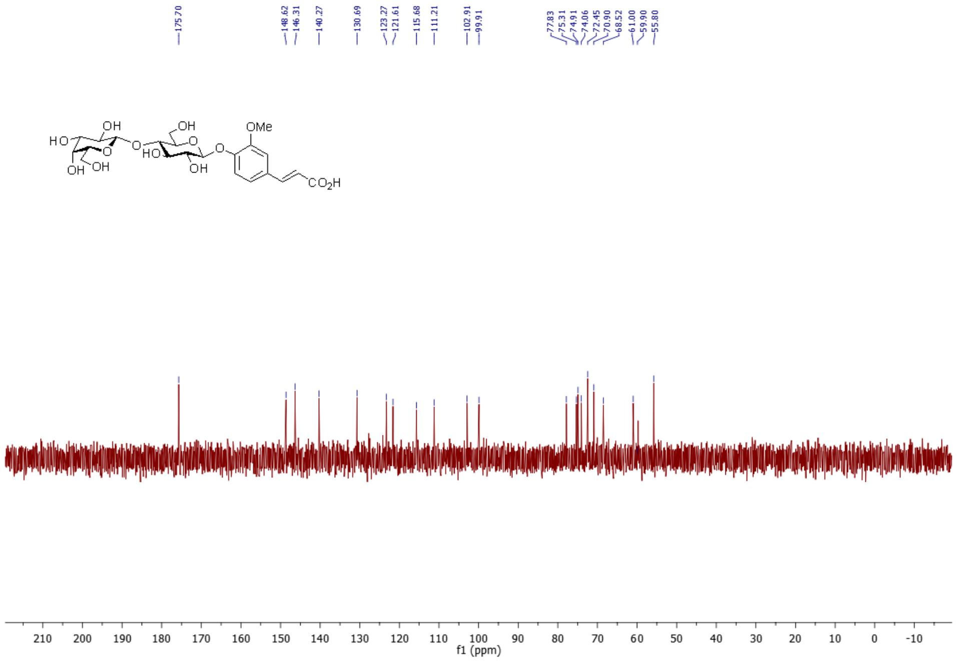

^{13}C NMR spectrum (75MHz, D_2O) of compound **18**.

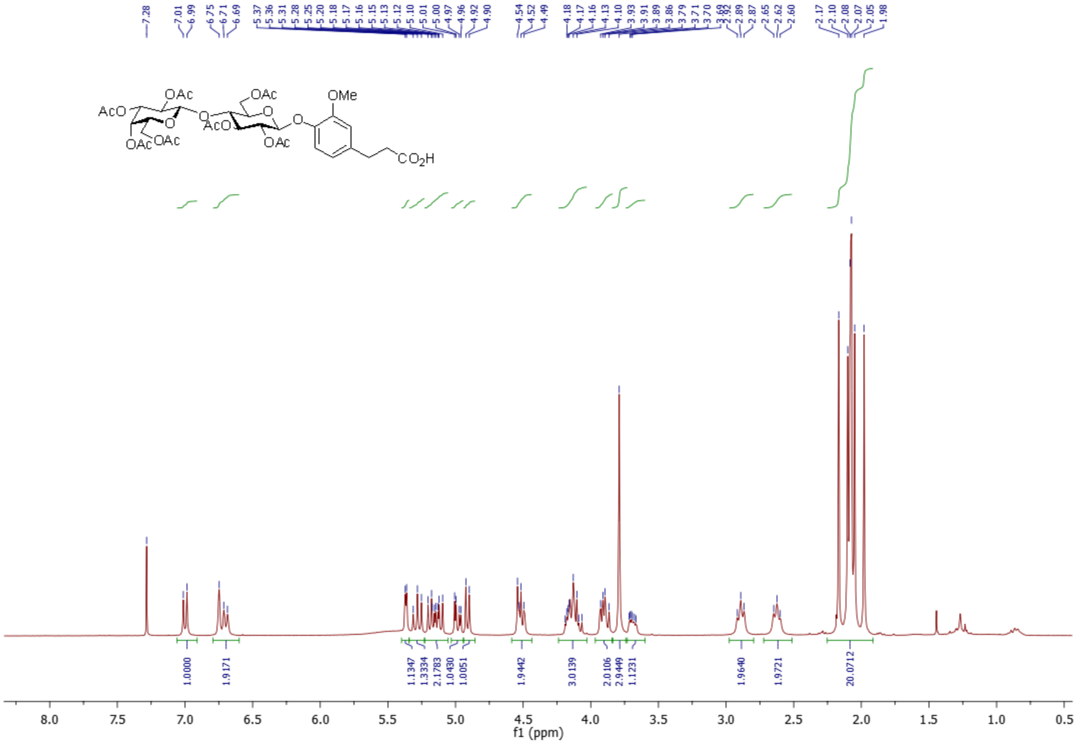

^{1}H NMR spectrum (300 MHz, $CDCl_3$) of compound **19**.

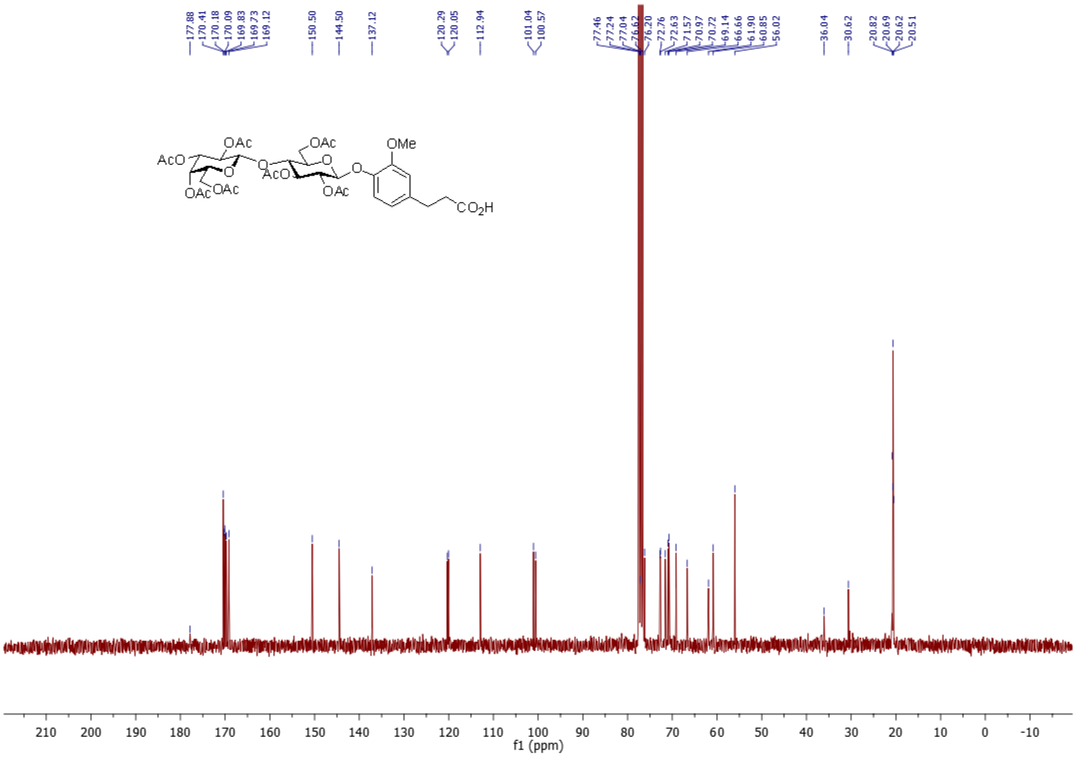

^{13}C NMR spectrum (75 MHz, $CDCl_3$) of compound **19**.

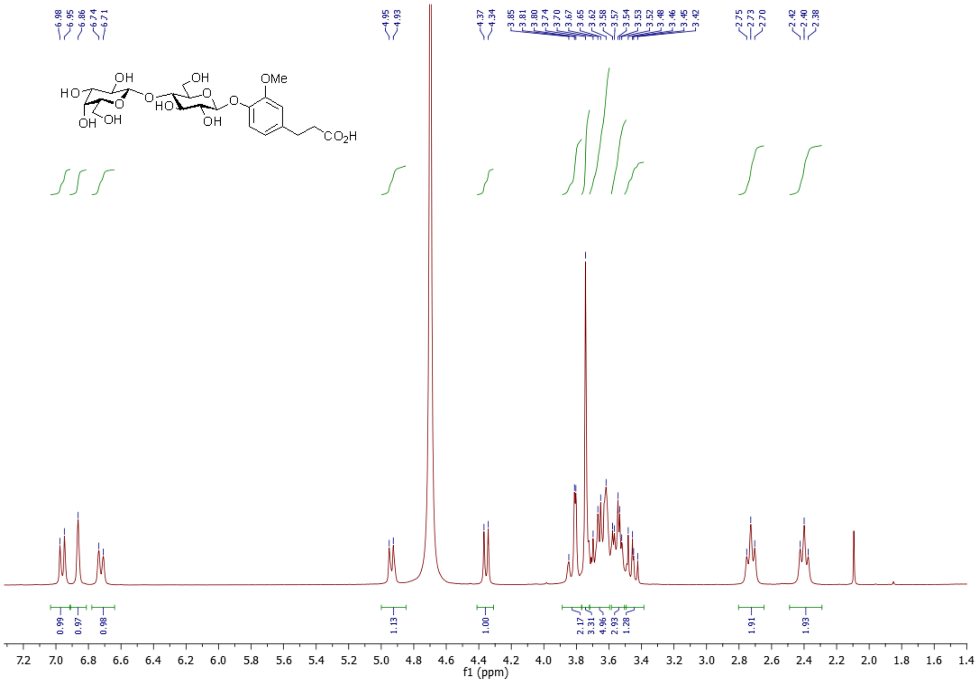

^{1}H NMR spectrum (300 MHz, D_2O) of compound **20**.

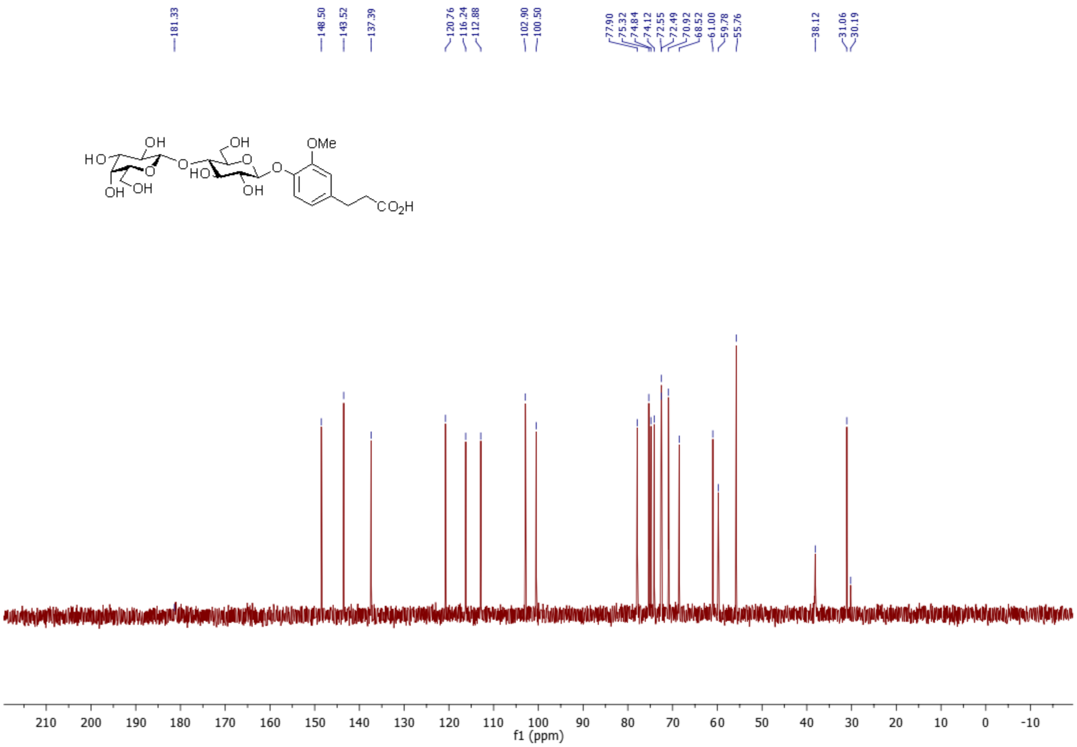

^{13}C NMR spectrum (75 MHz, D_2O) of compound **20**.

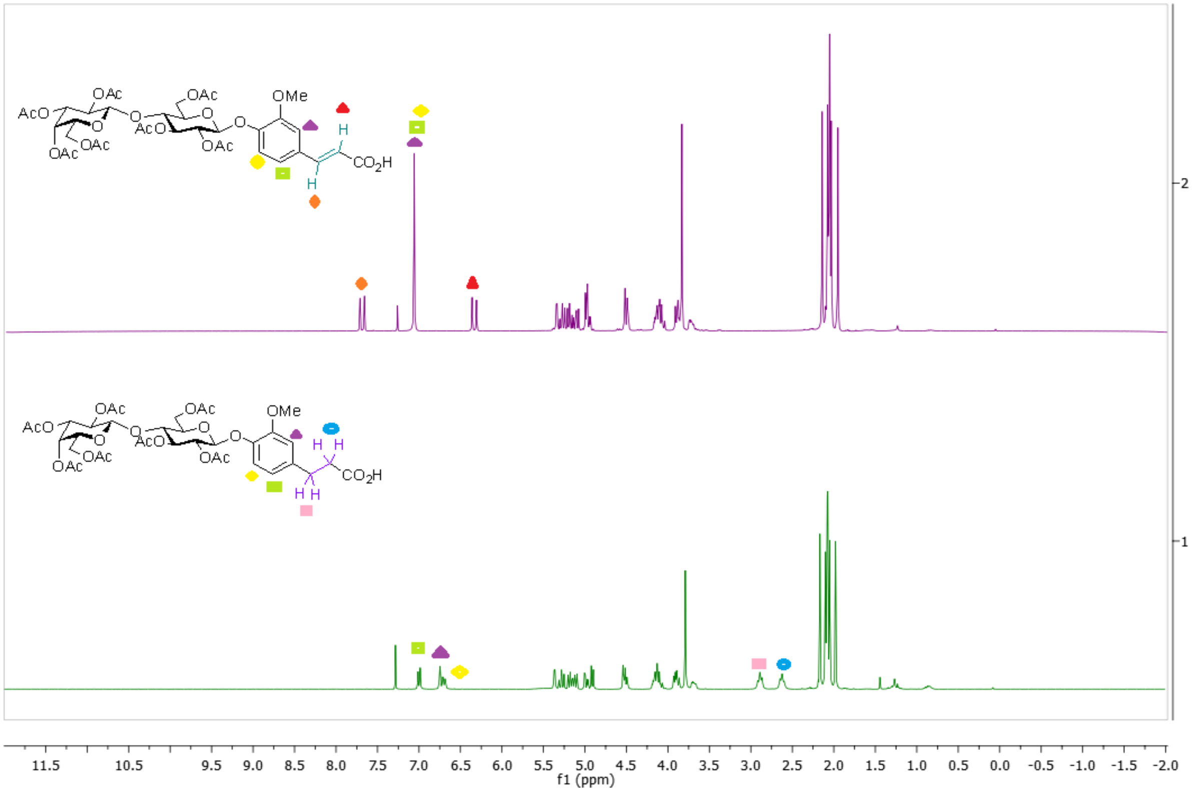

Comparison of ^{1}H NMR spectra ($CDCl_3$, 300 MHz) of **17** and **19** with the appearance/disappearance of protons of the α,β-unsaturation.
